# Origin of Elevated S-Glutathionylated GAPDH in Chronic Neurodegenerative Diseases

**DOI:** 10.3390/ijms24065529

**Published:** 2023-03-14

**Authors:** Paul A. Hyslop, Leonard N. Boggs, Michael O. Chaney

**Affiliations:** 1 Arkley Research Laboratories, Arkley BioTek, LLC, 4444 Decatur Blvd., Indianapolis, IN 46241, USA; lboggs@arkleybiotek.com; 2 Eli Lilly Research Laboratories, Eli Lilly & Co., Lilly Corporate Center, Indianapolis, IN 46285, USA; chaney1643@comcast.net

**Keywords:** glutathione, oxidative stress, redox signaling, glyceraldehyde-3-phosphate dehydrogenase, hydrogen peroxide, neurodegenerative disease, molecular dynamic simulation

## Abstract

H_2_O_2_-oxidized glyceraldehyde-3-phosphate dehydrogenase (GAPDH) catalytic cysteine residues (C*_c_*(SH) undergo rapid S-glutathionylation. Restoration of the enzyme activity is accomplished by thiol/disulfide S_N_2 displacement (directly or enzymatically) forming glutathione disulfide (G(SS)G) and active enzyme, a process that should be facile as C*_c_*(SH) reside on the subunit surface. As S-glutathionylated GAPDH accumulates following ischemic and/or oxidative stress, in vitro/silico approaches have been employed to address this paradox. C*_c_*(SH) residues were selectively oxidized and S-glutathionylated. Kinetics of GAPDH dehydrogenase recovery demonstrated that glutathione is an ineffective reactivator of S-glutathionylated GAPDH compared to dithiothreitol. Molecular dynamic simulations (MDS) demonstrated strong binding interactions between local residues and S-glutathione. A second glutathione was accommodated for thiol/disulfide exchange forming a tightly bound glutathione disulfide G(SS)G. The proximal sulfur centers of G(SS)G and C*_c_*(SH) remained within covalent bonding distance for thiol/disulfide exchange resonance. Both these factors predict inhibition of dissociation of G(SS)G, which was verified by biochemical analysis. MDS also revealed that both S-glutathionylation and bound G(SS)G significantly perturbed subunit secondary structure particularly within the S-loop, region which interacts with other cellular proteins and mediates NAD(P)^+^ binding specificity. Our data provides a molecular rationale for how oxidative stress elevates S-glutathionylated GAPDH in neurodegenerative diseases and implicates novel targets for therapeutic intervention.

## 1. Introduction

Exposure of vulnerable cells to reactive oxygen species (ROS) such as H_2_O_2_ is being increasingly recognized as a common factor associated with the pathophysiology of vascular and neurodegenerative diseases. The redox signaling enzyme glyceraldehyde-3-phosphate dehydrogenase (GAPDH) is involved at multiple levels of redox signaling [1,2,3,4,5,6,7,8]. In a companion publication [9], we identified the mechanism through which H_2_O_2_ oxidizes GAPDH to a metastable signaling conformer via a two-cysteine switch. Initially, H_2_O_2_ selectively oxidizes the subunit catalytic cysteine residue C*_c_*(SH) followed by oxidation of the vicinal cysteine C*_v_*(SH) (four residues downstream) to their respective sulfenic acids C*_c,v_*(SOH). The two sulfenic acids rapidly and irreversibly condense to form an interchain sulfinic ester, inducing an intrachain conformational strain resulting in a new metastable subunit conformer, which we propose forms pro-apoptotic signaling complexes with chaperone proteins [9] that translocate to the nucleus and mitochondria (reviewed in [10,11]).

The major cellular antioxidant thiol, glutathione (G(SH)), reversibly reacts with C*_c_*(SOH) to form S-glutathionylated subunits, thereby blocking H_2_O_2_ oxidation of C*_v_*(SH) and the formation of the putative signaling conformer [9]. Reactivation of C*_c_*S-glutathionylated subunits is accomplished by either S_N_2 thiol-disulfide exchange by a second molecule of G(SH) or deglutathionylation by a family of G(SH) transferases such as glutaredoxin and thioredoxin. In a recent study [12], following H_2_O_2_ oxidation of *h*-GAPDH in vitro by the addition of 5 mM G(SH) in the absence and presence of glutaredoxin for 24 h, a modest increase in enzyme reactivation was observed compared to G(SH) on its own. In cellular models of redox stress, the formation of S-glutathionylated GAPDH and its subsequent reactivation has been reported [11,13,14,15,16]. S-glutathionylated GAPDH has been shown to translocate to the nucleus and initiate apoptotic signaling via the GAPDH/SitT1/p53 pathway [17]. S-glutathionylated GAPDH is also found in tissues following ischemia reperfusion or chronic neurodegenerative disease [1,18,19,20], and S-glutathionylated GAPDH in blood samples from patients with Alzheimer’s disease (but not healthy controls) was elevated and this correlates with severity and disease progression [21].

In our companion study [9], we noted that the bimolecular rate constant for H_2_O_2_ oxidation of C*_c_*(SH) located at the hydrophilic surface of the GAPDH subunits was no different from that of cysteine (~10 M^−1^s^−1^). The elevated C*_c_*S-glutathionylated GAPDH observed after H_2_O_2_ oxidative stress thus cannot be directly attributable to the enhanced reactivity of GAPDH C*_c_*(SH) to H_2_O_2_ and furthermore, subunit C*_c_*S-glutathionylation would be expected to be readily reactivated by S_N_2 thiol-disulfide exchange by both cellular G(SH) and by enzyme deglutathionylating G(SH) transferases.

For the above reasons, we designed this study with the primary goal of elucidating which factors might contribute to the persistence of S-glutathionylated GAPDH in various pathophysiological conditions. Using biochemical and in silico molecular dynamic simulations (MDS) with a subunit of the crystal structure of human GAPDH (1u8F), we established that the glutathione moiety of S-glutathionylated GAPDH at physiologically relevant intracellular conditions (pH 7, 37 °C and 1–5 mM G(SH)) undergoes S_N_2 displacement by a second glutathione molecule and this process is kinetically unfavorable compared with enzyme activation of S-glutathionylated GAPDH with small alkyl thiols such as β-mercaptoethanol. We demonstrate that this observation can be attributable to tight binding interactions between charged and hydrogen donor/acceptor atoms of both glutathione and glutathione disulfide (G(SS)G) and local residues of the GAPDH subunits within the active site pocket. Both S-glutathionylation and accommodation of G(SS)G within the active site results in significant reversable conformational subunit rearrangement with pronounced changes in the cofactor and protein binding S-loop region [22] of the subunit downstream from the active site. Given the central role of GAPDH in energy metabolism and redox signaling, developing a more complete understanding of the biochemical mechanisms giving rise to modified GAPDH with S-glutathione and bound G(SS)G and the consequences thereof may assist in the discovery of novel therapeutic interventions for both acute and chronic vascular and neurodegenerative diseases.

## 2. Results

### 2.1. GAPDH Assay

**Figure 1:**
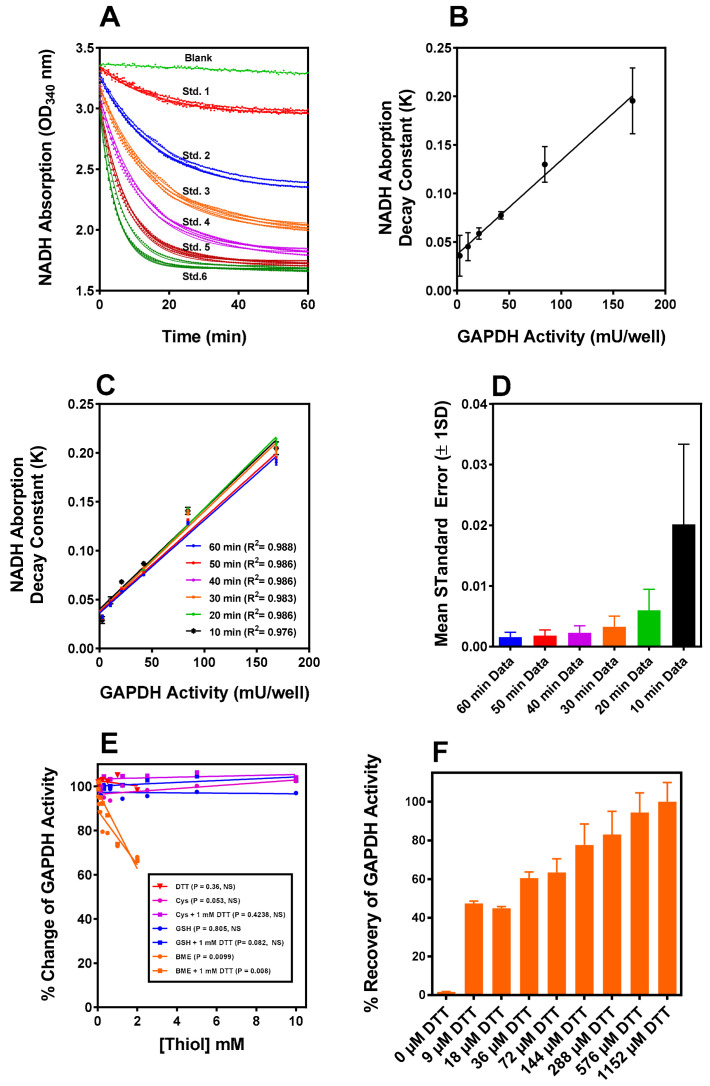
(**A**) The gluconeogenic enzyme GAPDH assay standard curve data generated by following the decrease in NADH absorption over a 60 min time window in the assay coupled to 3-PGA kinase phosphorylation of 3-PGA. The absorption decrease in the absence of GAPDH (blank) was first fitted using linear regression. The interpolated values at each timepoint were added to all concentrations of GAPDH data points from the standard curve and the samples to be measured. (**B**) First, all data sets were corrected for the time-dependent decrease in absorption of the blanks. The data were fitted to a monoexponential decay measured on three separate days. The rate constants calculated from the data in panel (**A**) are directly proportional to the amount of enzyme in the assay in the three experiments, and the slopes are not significantly different. (**C**) As a test of the validity of the data fit model, the 60 min data were truncated in steps of 10 min and the results each successive truncation plotted. (**D**) The SEM’s for the rate constants for each truncation obtained from the exponential fit successively declined from a maximum at 10 min to a minimum at 60 min, demonstrating that the data modeling is robust. The value of the exponential rate constant is independent of the assay start time, convenient for manual multiwell microtiter plate kinetic assays. (**E**) The four thiols used in this assay (DTT, BME, G(SH), and Cysteine) are examined for interference in the GAPDH assay. BME interference is observed and noted at concentrations used in the study. (**F**) IOB oxidized GAPDH is reactivated by DTT at various concentrations. Reactivation by 500 μM DTT does not significantly differ from full activation at 1 mM used to assess the % recovery of oxidized GAPDH under various experimental conditions.

GAPDH was assayed in this study using gluconeogenic substrates as the glycolytic substrate because (D or (DL) glyceraldehyde-3-phoshate (G3P) was unavailable from any vendor when we initiated the study. We encountered difficulty establishing reproducible linear “initial rates” of NADH oxidation in a microtiter plate format (kinetic and equilibrium barriers to substrate flux present in the gluconeogenic compared to glycolytic functions of GAPDH [23] contribute to assay complexity). Therefore, analytical modelling of dose response data for constructing standard curves were used and it yielded a monoexponential decay-to-plateau model, as shown in Figure 1A. The calculated NADH oxidation rate constants were directly proportional to the GAPDH concentration (Figure 1B) over the range required for the sample analysis and the slopes of the linear fit were not significantly different when measured over three separate days. Truncation of the data collection in 10 min segments from 60 min to 10 min did not significantly change the recalculated rate constants (Figure 1C), and their associated SEM was numerically the smallest at 60 min (Figure 1D), so 60 min was adopted as the standard assay time. The exponential data modelling is useful in a microtiter plate format as the NADH oxidation decay constant for all samples is independent of the relative initiation time of the assay. Using the assay parameters described above, measurements of the interference of the thiols referenced in this study (Figure 1E) revealed that G(SH), cysteine, and dithiothreitol (DTT), had no significant interference when observed over the concentration ranges, while a small ~10% interference was observed with ~1.13 mM β-mercaptoethanol (BME).

### 2.2. Iodosobenzoic Acid (IOB) Oxidation of GAPDH

The selective oxidation of the active site cysteine residues, C*_c_*(SH) in each subunit of the homotetramer of porcine GAPDH (*p*-GAPDH) results in thiol-reversible formation of stabilized sulfenic acids C*_c_*(SOH), with full recovery of dehydrogenase activity [24]. The use of IOB for selective stoichiometric oxidation of C*_c_*(SH) has been previously described [25]. Treatment of rabbit GAPDH (*r*-GAPDH) with IOB (8:1 mol IOB/mol *r*-GAPDH subunit) at RT for 10 min at pH 7.0 inactivated > 95% of dehydrogenase activity. Restoration of dehydrogenase activity following the removal of excess IOB by spin column buffer exchange (SCBE) was observed to be ~95% after incubation of the oxidized enzyme for at least 1 h following the initiation of oxidation, or after a freeze-thaw cycle prior to incubation with 1 mM DTT for 15 min (requirements for use of this concentration of DTT to achieve maximal enzyme reactivation are shown in Figure 1F).

### 2.3. Reaction of IOB-Oxidized GAPDH with G(SH)

After incubation of IOB-oxidized *r*-GAPDH with 1.2:1 mol G(SH)/mol GAPDH subunit for 10 min measurement of S-glutathionylated subunits using the Promega Glo assay (Methods and Materials) resulted in approximately four (3.7 ± 0.23, *n* = 4) mol G(SH)/mol. 100% recovery of GAPDH enzyme activity was observed after 15 min incubation with DTT (Figure 1A green bar), and as with the IOB-oxidized enzyme, recovery of the S-glutathionylated enzyme activity after DTT activation was not significantly different compared to the reference native GAPDH activity obtained prior to freeze-thaw.

**Figure 2:**
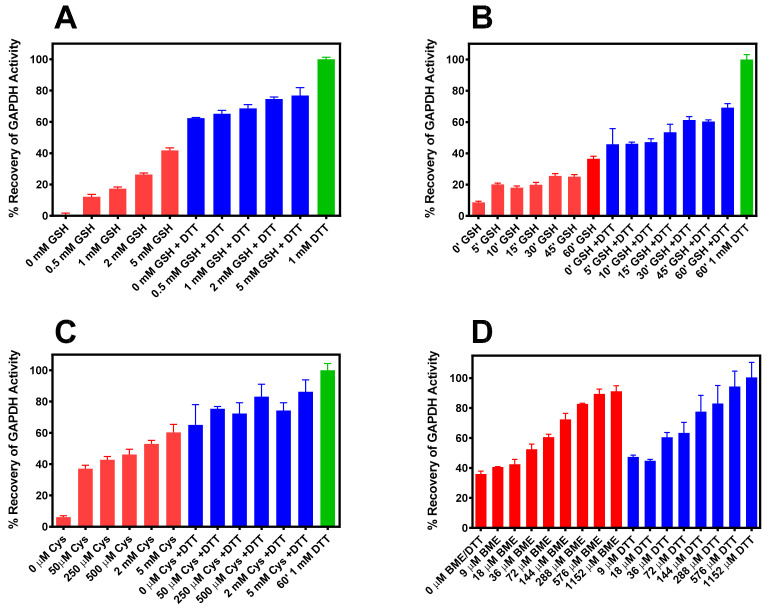
(**A**) Dose response for reactivation of S-glutathionylated GAPDH by G(SH) in the absence (red bars) and presence of 1 mM DTT (blue bars). (**B**) Time course of reactivation of S-glutathionylated GAPDH by 1 mM G(SH) in the absence (red bars) and presence of 1 mM DTT (blue bars). (**C**) Dose response of reactivation of S-cysteinylated GAPDH by cysteine in the absence (red bars) and presence of 1 mM DTT (blue bars). (**D**) Dose response of reactivation of S-mercaptoethanolylated GAPDH by either BME in the absence (red bars) and DTT (blue bars). The data (Mean ± SD, n = 3) are from representative experiments. The green bars in Panels **A**, **B** and **C** represent incubation without G(SH) in the presence of 1 mM DTT.

Recovery of the dehydrogenase activity of S-glutathionylated *r*-GAPDH was demonstrated when incubated in the presence of 0–5 mM G(SH) in the presence or absence of 1 mM DTT for 15 min prior to the GAPDH assay (Figure 2A, red bars). The ability of glutathione to reactivate the dehydrogenase activity of the S-glutathionylated enzyme was augmented compared with DTT (Figure 2A, blue bars). At the highest concentration of G(SH), 42 ± 1.6% dehydrogenase activity was recovered compared with the activation of the IOB oxidized enzyme by 1mM DTT alone (Figure 2A, green bar). Surprisingly, the same post S-glutathionylation concentrations of G(SH) also impaired the ability of 1 mM DTT to dose-dependently reactivate the enzyme, as post-incubation with 5 mM G(SH) and 1 mM DTT only restored 65.2 ± 2.1% to that of DTT alone (Figure 2A, blue bars).

The addition of 1 mM G(SH) ± 1 mM DTT following S-glutathionylation of oxidized GAPDH was assessed at various time intervals for 1h. The data in Figure 2B shows that incubation with G(SH) ± 1 mM DTT over 60 min resulted in a time-dependent increase in recovery of dehydrogenase activity. The kinetics of post S-glutathionylated enzyme activity recovery in the presence of 1 mM G(SH) ± DTT was sluggish, such that at 60 min, only 35.6 ± 2.6% and 69.1.6% was observed ± DTT, respectively. The approximate intracellular physiological conditions under which reduction of S-glutathionylated of GAPDH by G(SH) would be expected should be in the range of ~ 0.5–5 mM G(SH), pH 7, 37 °C. This demonstrates that, at least with respect to the major intracellular thiol-disulfide S_N_2 exchange reaction by G(SH), GAPDH glycolytic activity is compromised after H_2_O_2_ oxidative stress by slow S-deglutathionylation. Since the low-mw dithiol, DTT was less effective at reactivating S-glutathionylated enzyme in the presence of G(SH) than in its absence, this indicates that steric factors may be responsible for these observations.

### 2.4. Reaction of IOB-Oxidized GAPDH with Cysteine

Qualitatively, similar results were observed following S-cysteinylation of IOB oxidized GAPDH. However, in a post incubation experiment for 15 min with varying concentrations of cysteine ± DTT recovery of S-cysteinylated GAPDH activity was more effective than that observed with G(SH) (Figure 2C). Incubation with 5 mM cysteine restored S-cysteinylated enzyme activity by 60.3 ± 5.15% (Figure 2C red bars. When 1 mM DTT was included in the incubation with cysteine 86.2 ± 2.6% of dehydrogenase activity were recovered (Figure 2C blue bars).

### 2.5. Reaction of IOB-Oxidized GAPDH with BME and DTT

A dose response study using S-mercaptoethanolated GAPDH was performed with a 15 min. post-incubation with various concentrations of BME or DTT prior to assay (Figure 2D). Recovery of GAPDH activity was significantly increased at lower concentrations of BME or DTT than with either G(SH) or cysteine. 1.152 mM BME (Figure 2D, red bars) restored enzyme activity to levels expected allowing for the small inhibitory effect of BME at the highest concentration observed in Figure 1E, while DTT (Figure 2D blue bars) fully restored GAPDH. A 50% reactivation of enzyme activity was achieved at ~36 μM by both BME and DTT. These latter experiments confirm that both S-glutathionylated and S-cysteinylated GAPDH are resistant to reduction by the two ubiquitous intracellular thiols, G(SH) and cysteine. This observation is particularly relevant to G(SH), because S-glutathionylated GAPDH has been consistently observed in pathological conditions as noted in the introduction section.

### 2.6. Molecular Dynamic Simulations of G(SH) Docking in the Active Site of Oxidized GAPDH

**Figure 3:**
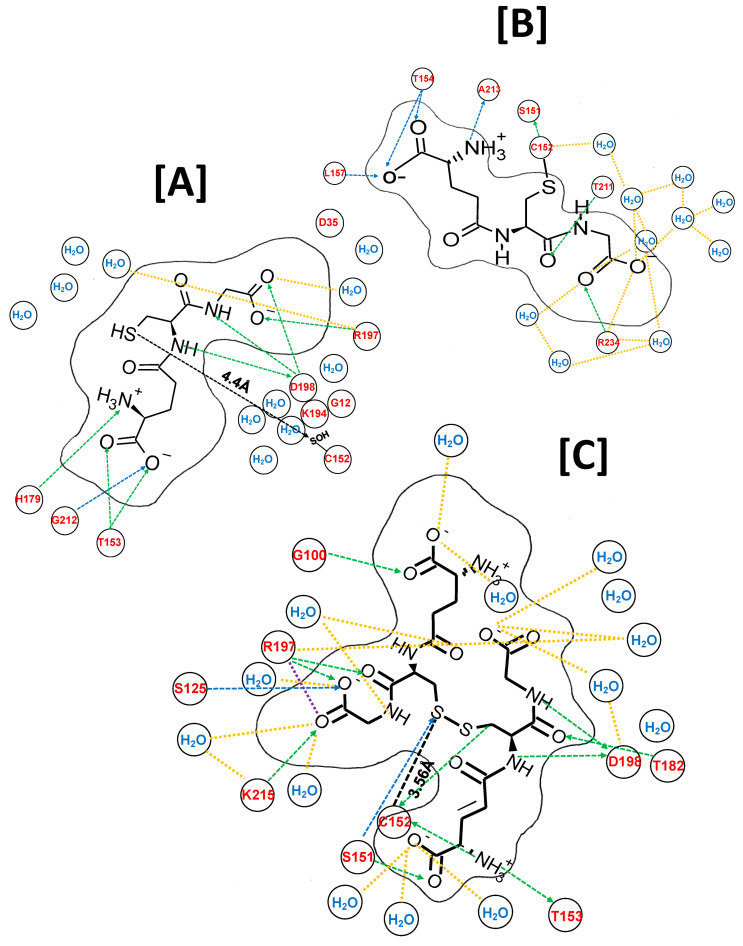
MDS ligand interactions within and around the active site of an isolated subunit of solvated *h*-GAPDH (1u8F) represented in two dimensions. (**A**) A catalytic cysteine residue (C152(SH) GAPDH subunit is converted to C*_c_*(SOH), the initial step of the oxidation of GAPDH by H_2_O_2_ and a molecule of G(SH) is added to the model. After MDS, the G(SH)-C152(SH) sulfur-sulfur are separated by 4.4 Å, within range of the disulfide bond formation. The H-bond donor and acceptor network are shown in green and blue dotted lines, and the bond lengths are obviously distorted by a 2D rendering. The atomic bond distances and interaction energies are tabulated in Supplementary Tables S1 and S2. The solid black line delineates the van der Walls molecular cross section of G(SH). (**B**) S_N_2 nucleophilic attack by G(SH) on sulfenic acid is simulated by the formation of the disulfide bond in MOE, forming C152S-glutathione. (**C**) A second molecule of G(SH) is placed within the active site pocket, and after MDS it is docked in an antiparallel alignment with C152 S-glutathione. S_N_2 nucleophilic attack by G(SH) on the S-glutathionylated structure forms a docked G(SS)G ligand and reducesC152(SH) with their nearest sulfur-sulfur distance of 3.56 Å, such that the three sulfurs atoms can undergo thiol-disulfide exchange resonance.

The crystal structure of an isolated subunit of human GAPDH (*h*-GAPDH) PDB 1u8F was used for in silico analysis, with C152(C*_c_*SH) converted to C152(SOH) using the MOE builder utility. A molecule of glutathione (ligand) was placed in the vicinity of C*_c_*152(SOH), and energy minimized. After 100 ps of molecular dynamics, the structure was annealed, and energy minimized (MDS). The resulting ligand-receptor interactions, the type of interaction, with atomic distances and interaction energies (ΔE) for NAD^+^ and G(SH) are listed in Supplementary Tables S1A and S2A, top panel. The 2D representation of the docked ligand is shown in Figure 3A. The relationship between the ligand-receptor value of ΔE and its binding affinity (K*_a_*) is complex (the value of ΔE computed within MOE is a thermodynamic quantity (Methods and Materials) whereas K*_a_* is measured directly from the kinetic equilibrium (K*_a_* = k_on_/k_off_). It is useful to compare ΔE obtained for G(SH) with that of the NAD^+^ cofactor obtained using the same MOE algorithms within the native subunit as a comparator shown in Supplementary Table S1A. The intracellular concentrations of G(SH) and NAD^+^ are within the same order of magnitude and both values of ΔE are comparable and the values of K*_a_* for NAD^+^ are between 10^−11^ and 10^−5^ M (see Ref. [26]). This indicates that G(SH) is tightly bound within the active site of the enzyme.

The introduction of G(SH) (ligand) into the active site increased the total ΔE for NAD^+^ (ligand) and GAPDH between the non-bonded ligand hydrogen atoms and atoms of the local side chains, the polypeptide backbone of the subunit, and solvent increased from −60.5 kcal/mol to −73.2 kcal/mol (Supplementary Tables S1B and Table 1. The G(SH) molecule docked with its sulfur atom at a distance of 4.4 Å (Figure 3A) from the sulfur atom of C152(SOH), within van der Wall’s radius for disulfide bond formation ≤ (4.9 Å). The individual ligand bonding energies are shown in Supplementary Table S2A, top panel, with the total ΔE = −77.2 kcal/mol (Table 1). These factors indicate that the docking of glutathione within the proximity of C*_c_*152(SOH) orients the glutathione sulfur atom for nucleophilic attack on C152(SOH) for the formation of mixed disulfide.

### 2.7. Molecular Dynamic Simulations of Atomic Interactions of S-Glutathione within the Active Site

In the next step, the disulfide bond between the docked glutathione sulfur and C*_c_*(SOH) sulfur was formed within the MOE builder utility and was subject to a second round of MDS and energy minimization. The resulting NAD^+^ and S-glutathione moiety interaction binding energies within the active site are shown in Supplementary Tables S1C and S2A, bottom panel, respectively. The total ΔE for NAD^+^ docking (Table 1) decreased to −43 kcal/mol, while the total ΔE docking for the docked S-glutathione moiety was 44.3 kcal/mol (Table 1). The 2D representation of the interactions between the S-glutathione moiety and the active site region is shown in Figure 3B.

### 2.8. Molecular Dynamic Simulations of the Atomic Interactions within the Active Site after Introducing a Second Molecule of G(SH)

A second molecule of glutathione was readily accommodated within the locality of the S-glutathionylated subunit within MOE. After MDS and energy minimization as before, the two glutathione molecules aligned in an anti-parallel formation with respect to each other, with their sulfur centers at 3.56Å, within the van der Waals distance for disulfide bond formation. The G(SH) and the S-glutathionylated moiety and active site residue individual bond ΔE values are listed in Supplementary Table S2B in the top and bottom panels, respectively. The individually calculated total ΔE for the docking of G(SH) and for local interactions for the S-glutathione were 55.0 kcal/mol and 68.1 kcal/mol, respectively (Table 1).

### 2.9. Molecular Dynamic Simulations of the Atomic Interactions within the Active Site after Breaking the GAPDH S-Glutathione Mixed Disulfide Bond and Formation of Cystine (G(SS)G

The S-glutathione disulfide bond was broken and reformed between the two G(SH) molecules, simulating the S_N_2 displacement reaction. This was followed by a third round of MDS and energy minimization. The resulting 2D structure is shown in Figure 3C. The types of interaction, with atomic distances and interaction energies for NAD^+^ and G(SS)G) are shown in Supplementary Tables S1D and S2C, respectively. The calculated total ΔE for NAD^+^ was −44.1 kcal/mol, while the ΔE for the docked G(SS)G ligand and local amino acid residues was −85.3 kcal/mol (Table 1).

The sulfur-sulfur distances between the proximal G(SS)G sulfur atom and C*_c_*152 sulfur atom was 3.56 Å, well within the van der Wall’s sulfur-sulfur covalent bonding distance (Figure 3C). These observations indicate that the docked G(SS)G and C*_c_*152 can theoretically participate in S_N_2 disulfide exchange resonance. The combination of the G(SS)G binding interactions and thiol-disulfide exchange are likely to inhibit dissociation of G(SS)G from the subunit active site and provides an explanation for why G(SH) is a relatively poor reactivator of S-glutathionylated GAPDH.

### 2.10. Molecular Dynamic Simulations of Cysteine Docking in the Active Site of Oxidized GAPD

If atomic interactions within the active site of GAPDH participate in the slow deglutathionylation process (at least by a thiol-disulfide exchange mechanism), then if the relative atomic interactions within the active site of the enzyme after accommodation of a cysteine molecule follow a similar pattern as G(SH), this would add credence to the hypothesis.

Because of time and resource constraints, fewer parameters were explored with cysteine than with G(SH). After placement of a cysteine molecule within the active site region of *h*-GAPDH with C152 oxidized to C152(SOH), following a round of MDS and energy the total binding interactions between cysteine and atoms within the active site was −22.6 kcal/mol/ (Supplementary Table S3A). After forming the mixed disulfide bond within MOE, the total interaction energies between the S-cysteinylated moiety and the local active site atoms were −23.8 kcal/mol (Supplementary Table S3B). After breaking the S-cysteinylated mixed disulfide bond and forming cystine (C(SS)C), the atomic interaction energies between the dipeptide and local active site atoms increased to −97.1 kcal/mol (Supplementary Table S3C), indicating that as with G(SS)G), C(SS)C was also tightly bound within the active site of GAPDH.

### 2.11. Measurement of Time-Dependent S-glutathionylated and Non-Covalently Bound G(SH) and G(SS)G to GAPDH

To provide biochemical support for the above conclusions drawn from MDS calculations, IOB oxidized S-glutathionylated *r*-GAPDH was incubated with 1 mM G(SH) for a total 75 min, and samples withdrawn from the incubation very 15 min. Samples were analyzed after SCBE to remove non-GAPDH associated G(SH) and G(SS)G). Denaturation of the sample in the upper chamber of the Microcon^®^ ultrafiltration device allows centrifugal separation of the S-glutathionylated GAPDH remaining in the retentate, while Non-Covalently bound G(SH) and G(SS)G associated with GAPDH can flow through into the eluate. The ratios of oxidized and reduced G(SH) in the samples were then assessed using the Promega Glo assay described in Methods and Materials.

**Figure 4:**
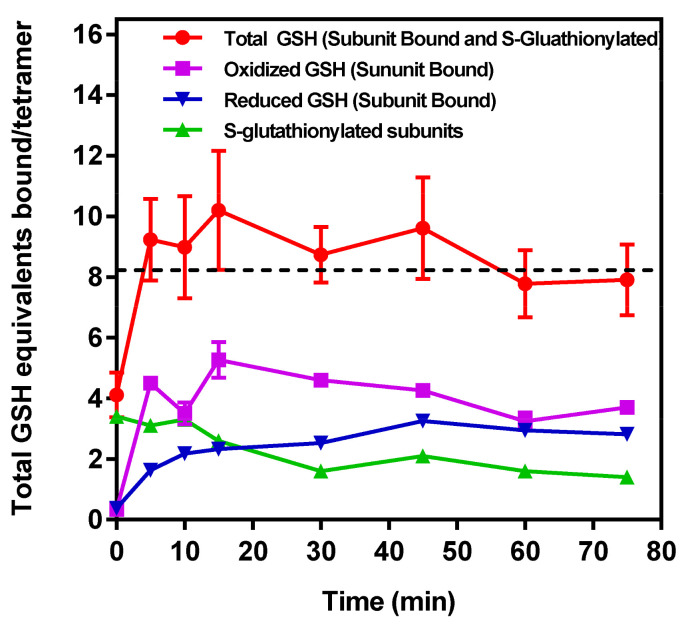
Distribution of S-glutathionylated and bound G(SH) and G(SS)G as a function of time of incubation with 1 mM G(SH). G(SH) and G(SS)G were measured using the Promega oxidized/reduced protocol and reagent kit. (a 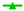) Time course of the decline in total mol of S-glutathionylated subunits GAPDH/mol tetramer measured following the removal of all unbound G(SH) and G(SS)G after subunit denaturation and washing in the retentate following Microcon® spin separation (see text for details). (b 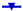) Time course of unbound reduced G(SH) recovered and measured in the eluate following Microcon® spin separation. (c 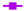) Time course of unbound G(SS)G recovered and measured in the eluate following Microcon® spin separation. Note: the data represents 2 mol equivalents of G(SH) derived from 1 mol equivalent of G(SS)G in the Promega protocol). (d 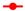) The total number of G(SH) equivalents bound to GAPDH over the time course of the incubation of S-glutathionylated GAPDH with 1 mM G(SH) is shown as the sum of measurements. (a), (b), (c) The associated cumulative SD of the triplicate samples. The black dashed line represents the theoretical maximal G(SH) binding capacity of the four active sites within the GAPDH tetramer. The data show the combined mean values ± SD from two separate experiments with technical triplicates.

The results are shown in Figure 4 and the measured versus calculated values for the parameters are described in the figure legend. The S-glutathionylation of GAPDH subunits declined over time, whereas bound G(SH) and G(SS)G increased after ~10–15 min incubation with 1 mM G(SH) and then remained relatively constant over the time course of the experiment, which is reflected in the total bound [G(SH)] (Figure 4 top red filled circles), and is calculated as follows: (i) denatured S-glutathionylated GAPDH in the washed retentate and (ii) plus total [G(SH)] + 2 × [G(SS)G] recovered in the eluate. If the assumption is made that the enzyme activity is completely abolished when each GAPDH subunit active site harbors two molecules of G(SH (or 1 molecule of G(SS)G), it is enzymatically inactive; this assumption is consistent with the data obtained in Figure 1A,B. The experimental data indicate that when subunit active sites harbors a tightly bound molecule of G(SS)G or two molecules of tightly bound G(SH) the enzyme activity is inactivated. This may also be true of a single molecule of bound G(SH) in the native enzyme, although this seems unlikely as incubation of native GAPDH with up to 10 mM G(SH) (Figure 1E) had no effect on enzyme activity.

This was also true when the native enzyme was pre-incubated with 1 mM G(SS)G for 60 min and was assayed with 1 mM G(SS)G in the assay buffer (Supplementary Figure S1), indicating that G(SS)G cannot bind to the native enzyme, so presumably the conformational rearrangement induced when G(SS)G forms within the active site allows for G(SS)G to dissociate and leave, but not to re-enter when the subunit is restored to its native state.

### 2.12. Comparison of Secondary Structural Motifs of S-Glutathionylated GAPDH

**Figure 5:**
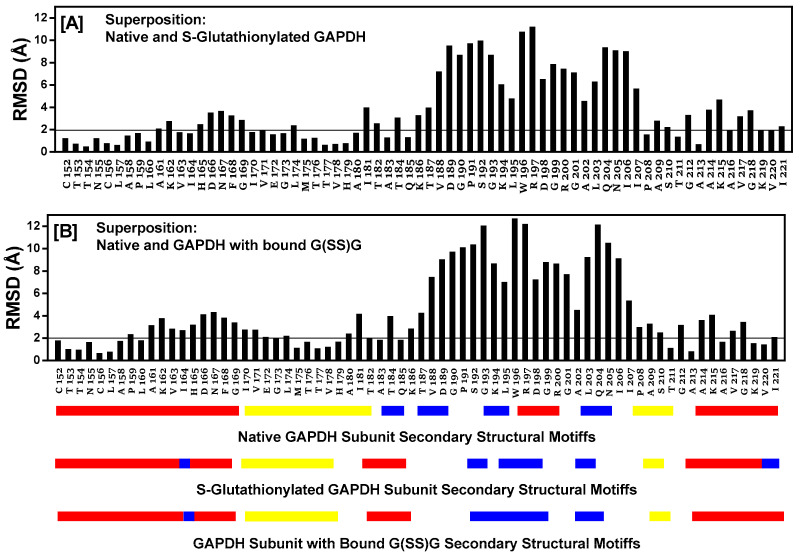
The protein sequence alignment and structure superposition tool in MOE is used to align the native *h*-GAPDH subunit structure with the output from MDS, and the energy minimizes subunit structures for C152S-glutathionylated subunit (Panel [**A**]) and the native *h*-GAPDH subunit structure with the output from MDS and the energy minimizes subunit structure for G(SS)G subunit docked within the active site (Panel [**B**]). The figure shows a truncated version of the full 335-amino acid sequence data shown in Supplementary Figures S2 and S3, to emphasize the region of greatest perturbation and includes all residues in the S-loop region of the subunit (residues 180–203). The root mean square distance (RMSD) for each alignment column (i.e., residue pair) used during the superposition. The RMSD value is represented by a black vertical bar above the pairwise aligned sequences. Closely matching RMSD values are highlighted by a horizontal line cutoff below 2.0 Å, while a larger RMSD excursion (5–13 Å) indicates poor atomic coordinate superposition. The linearized secondary structural features the following: Red α-helix; yellow β-sheet; and blue 2-5 residue turns; while no color is random coil for the three structure superpositions shown below Panel [**B**].

**Figure 6:**
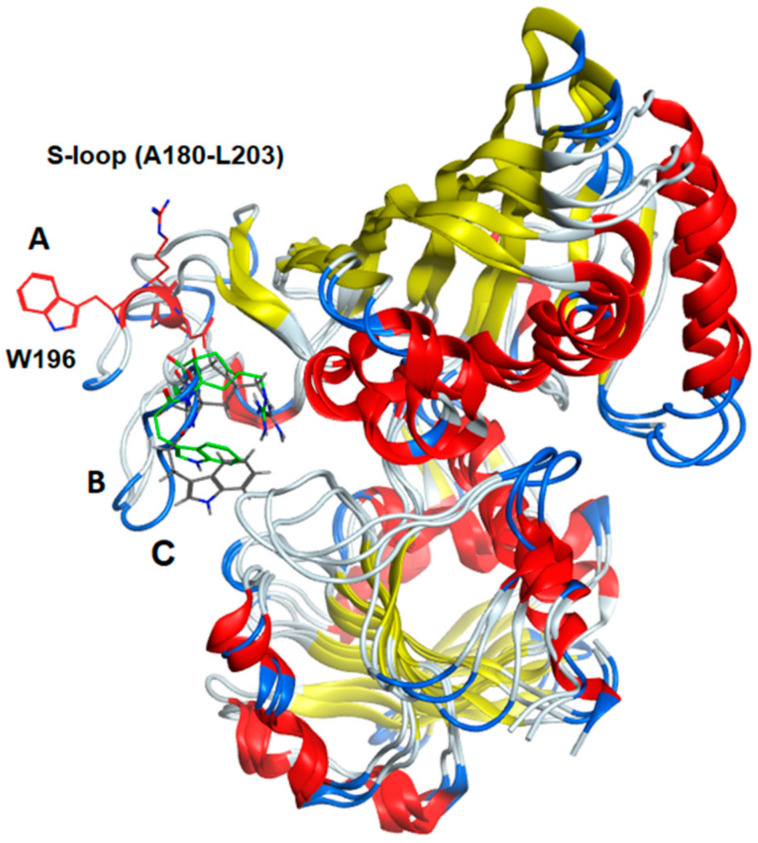
Ribbon diagram of the superimposed subunit structures of GAPDH (**A**) native, (**B**) S-glutathionylated, and (**C**) a subunit with tightly bound G(SS)G in the active site. The figure shows the clear transposition of the S-loop region (residues A180–L203) using of the two modified subunits compared to the native enzyme. The indole side chain of tryptophane W196 is shown as a visual marker for the residue displacement (native subunit is red, S-glutathionylated subunit is green, subunit with G(SS)G bound is black). The short α-helix (W196–R200) in the native subunit is converted to coil and turns in the modified subunits. The S-loop region is of interest because it is a site of protein binding and also mediates NAD(P)^+^ cofactor binding selectivity.

The entire subunit secondary structure of an S-glutathionylated subunit and one with G(SS)G) bound to the active site of *h*-GAPDH were compared to that of a native subunit. GAPDH subunits are broadly characterized as comprising two domains. The full 2D sequence alignment of the structural motifs (α-helix, β-sheet, turns, and random coil) are shown linearized in Supplementary Figure S2 (NAD^+^ domain) and Supplementary Figure S3 (catalytic domain). A common distinct feature of the comparisons with the native enzyme shows that several α-helical domains within the subunit are interrupted with turns. The results of the sequence alignment superposition matrix of the pairwise RMSD (root mean square distance) of the relative α-carbon atom coordinates showed modest (4–6 Å) perturbations within the NAD^+^ binding domain. A major perturbation (7.5–13 Å) was observed within the S-loop (residues 180–203) of the catalytic domain, presumed to interact with cellular proteins [22], and it is also responsible for conferring NAD(P)+ selectivity [27]. Truncated data from Supplementary Figure S3 are shown in Figure 5 for emphasis, and the overall structural ribbon superpositions of the subunit are shown in Figure 6, depicting the S-loop region, showing the short α-helix (W196–G199) in the native subunit converted to turns in the two other superpositions. The calculated averaged RMSD values for all of the amino acids in the subunits perturbed by S-glutathionylation when compared with the native was 2.72 Å, while subunits perturbed by the accommodation of bound G(SS)G was 3.06 Å. The calculated averaged RMSD values for all amino acids comparing the perturbations of subunits S-glutathionylated and G(SS)G decreased to 1.36 Å. While there are secondary structural differences induced by S-glutathionylated subunits and subunits with bound G(SS)G from the inspection of Supplementary Figures S2 and S3, a commonality they share is that they both induce a major perturbation in the S-loop region, evident in Figure 6.

## 3. Discussion

Full recovery of the enzyme activity of S-glutathionylated GAPDH was achievable with excess DTT after a 60 min incubation at 37 °C, pH 7, both before and after a freeze-thaw cycle, indicating that any subunit secondary structural perturbations induced by S-glutathionylation or by the accommodation of G(SS)G within the active site pocket were reversible. We used iodosobenzoic acid (IOB) to selectively oxidize C*_c_*(SH) [25], rather than H_2_O_2_, to avoid the complications introduced by more complex oxidation mechanisms of C*_v_*(SH) and the associated irreversible enzyme activation [9]. The relative ability of thiols to partially reactivate GAPDH activity oxidized with H_2_O_2_ was first observed to be in the order of G(SH) >>cysteine >BME/DTT [28], and our data are consistent with this result and serve to illustrate that full enzyme activity is recoverable when IOB rather than H_2_O_2_ is used to oxidize C*_c_*(SH).

The interaction of the S-glutathionylated moiety in GAPDH from *Arabidopsis thalania* with local active site residues was first noted by Zafaginini et al. [29] using infusion of GAPDH crystals with H_2_O_2_ and G(SH), and again with the caveat that H_2_O_2_ was used rather than a selective oxidizing agent, provides support for our ab initio MDS analysis and interpretation of S-glutathionylated *h*-GAPDH. An intriguing observation revealed by MDS is that following S_N_2 displacement of S-glutathione by a second molecule of G(SH), inhibition of the dissociation of G(SS)G from the active site may be augmented by the additional binding interactions of the glutathione hexapeptide, and also by the proximity of its sulfur atom closest to C*_c_*152, creating a thiol-disulfide exchange resonance between the protein and the hexapeptide atoms. While the limitations of interpreting ΔE in terms of binding affinity are well known, the value obtained for the G(SS)G interaction with the active site of GAPDH (~90 kcal/mol) indicates relatively tight binding. We demonstrate that the results and conclusions drawn from the MDS data are recapitulated experimentally in vitro, as S-glutathionylation of *r*-GAPDH incubated with 1 mM G(SH) reduced S-glutathionylated subunits over a 75 min incubation with the appearance of non-covalently bound G(SS)G and G(SH), as predicted.

Reversible secondary structural changes induced by S-glutathionylation were first demonstrated by Barinova et al. [12] through measurement of the relative thermal stabilities. Again, H_2_O_2_ was used as the oxidizing agent and so the complexity of the interpretation of the data with the native enzyme must be considered. However, smaller, but significant, structural perturbations were also observed in the same study when the C156S mutated enzyme was oxidized by H_2_O_2_ in the presence of G(SH) (where H_2_O_2_ oxidation of C156 is absent), providing a more direct biochemical validation of our MDS data.

GAPDH modified by S-glutathionylation and bound G(SS)G) are of interest because of the potential for the stabilized modified subunits to interact with cognate partner proteins involved in redox signaling. An analysis of the secondary structure and pairwise amino acid alignment of native *h*-GAPDH and modified with either S-glutathione or bound G(SS)G within the active site region revealed that significantly large perturbations in the secondary structure were especially apparent in the S-loop region (residues A180–L203) containing the short α-helix (W196–R200). The large perturbed relatively unstructured loop protrudes out from the center of the subunits and thus perturbation in this region is a potential site for binding the intracellular cognate partner proteins involved in the redox signaling pathways [22].

On the one hand, S-glutathionylation of partially oxidized GAPDH by H_2_O_2_ buffers further H_2_O_2_ oxidation of C*_v_*(SH), preventing irreversible enzyme activation and the adoption of its redox signaling conformer [9]. On the other hand, S-glutathionylation of GAPDH is comprised of glycolytic flux. In a cellular model of H_2_O_2_ oxidative stress, inhibition of glycolysis was observed to contribute to the depletion of intracellular ATP, which was almost entirely accounted for by GAPDH inactivation [30]. The cellular inactivation was a result of both irreversible and reversible (S-glutathionylation) enzyme inactivation, as well as of a decrease in NAD^+^ [31,32,33]. The physiological significance of S-glutathionylated GAPDH and GAPDH with bound (G(SS)G identified in this study is difficult to evaluate and obviously requires further investigation. In addition, it is likely to be dependent many factors, including the type and of tissue, species and duration of redox stressor (other ROS, nitric oxide etc.), prevailing intracellular G(SH) levels, relative contributions of enzymatic deglutathionylation, and almost certainly many other factors.

The persistence of S-glutathionylated GAPDH has consistently been observed in cellular models of oxidative stress and in tissues from chronic degenerative diseases (introduction). Neurons are particularly dependent on both extracellular glucose and glial-derived glucose and lactate to provide just enough metabolic energy to meet the demand. Inhibiting glycolysis at the triose phosphate step reduces the overall cellular energy charge [30,34] by decreasing mitochondrial substrate availability and by the non-productive consumption of ATP following the phosphorylation of glucose transported into the cell [30]. This is significant because falling neuronal ATP levels disrupt both cytoskeletal organization [35] and integrin-mediated adhesion to the extracellular matrix [36], processes that may contribute to initiating neurodegenerative disease. The pathophysiological reason(s) for the persistence of S-glutathionylated GAPDH is certainly worthy of further investigation, particularly because of its potential impact on vulnerable ischemia intolerant post-mitotic neurons. 

At least with respect to non-enzymatic deglutathionylation, our experimental results show (at least in part) the biochemical reasons why S-glutathionylated GAPDH is observed in the tissues and plasma in both acute and chronic degenerative diseases, and we postulate that enzymatic deglutathionylation may also be augmented for the same reasons, but this remains to be investigated and highlights the need to explore the origins and consequences of redox signaling for the identification of novel therapeutic targets to mitigate the devasting impact of chronic neurodegenerative diseases [3,5].

## 4. Methods and Materials 

### 4.1. GAPDH Sample Preparation and Oxidation

Rabbit GAPDH (Sigma; Cat No. G2267, St. Louis, MO, USA) was prepared fresh as needed for all of the experiments detailed in this work, using the methods below. First, the dry powder was reconstituted at ~1 mg/mL in 1 mM DTT and 1 mM NAD^+^ in deionized water and incubated for 30 min at room temperature. Then, the GAPDH samples were centrifuged at 20,000× *g* for 5 min at room temperature to remove any insoluble material. Next, to remove DTT, buffer exchange/desalting was accomplished using a 7000 MW cutoff Pierce Zeba^TM^ Spin Desalt Column resin (Pierce Biotechnology, Rockford, IL, USA), packed in columns, and used according to the manufacturer’s instructions. This method will be referred to from here on as Spin Column Buffer Exchange (SCBE). For all GAPDH studies, SCBE was performed using columns equilibrated with 50 mM K^+^-HEPES, 1 mM K-EDTA, and 0.1 mM NAD^+^ pH 7. Eluate protein concentrations free of DTT were measured using the Pierce (Rockford, IL, USA) BCA protein assay kit, adjusted to 1 mg/mL, and diluted 2.25-fold with K^+^-HEPES buffer containing a final eight-fold molar excess of the oxidant, iodosobenzoic acid (IOB). After mixing briefly and incubation for 10 min at room temperature, the resulting oxidized sample was again subjected to SCBE to remove the IOB and the protein concentration was determined.

### 4.2. Reduction of Oxidized GAPDH

For all the experiments exploring the reduction of oxidized GAPDH, the samples, prepared as described above, were further treated as follows, with either glutathione (G(SH)), L-cysteine (CYS), β-mercaptoethanol (BME), or dithiothreitol (DTT). All of these reducing agents were prepared as stocks in K^+^-HEPES buffer, added to oxidized GAPDH to attain the final molar ratio indicated in the figures, and then incubated for 15 min at 37 °C. In the experiments where further treatment or higher concentrations were desired, the reduced samples were further treated by adding 10× stock concentrations of reducing agents to thin-wall PCR tubes and quickly adding the undiluted reduced sample to dilute the agent to 1×. These tubes were mixed briefly then incubated at 37 °C for 10 min in wells of a metal heat block for all concentration response experiments and at the duration indicated for the time course experiments. Afterwards, these incubation samples were analyzed starting as quickly as possible using the kinetic assay described below.

### 4.3. Kinetic Assay for GAPDH

In order to determine the effect of treatments on GAPDH activity, we prepared a biochemical assay in which the β-NADH absorbance was monitored over time. GAPDH reduced the amount of β-NADH as it was consumed. Therefore, by comparing unknown samples to a standard sample (1 mM DTT and 1 mM EDTA, which was slow-frozen in aliquots at 0.5 mg/mL using 0.2% alkylated BSA [9] and 50 mM trehalose as the cryoprotectant) the specific activity of the GAPDH samples were interpolated. The GAPDH assay buffer (50 mM K^+^-HEPES, 130 mM KCl, 3 mM MgCl_2_, 1 mM K-EDTA, 1 mM potassium phosphate dibasic, 0.1% alkylated BSA, 8 mM 3-phosphoglyceraldehyde, 2 mM β-NADH, and 1.3 mM ATP, pH 7.0, measured at 37 °C) was prepared in a large batch without the 3-phosphoglycerol kinase (3-PGK) and frozen in aliquots. On the day of the assay, an aliquot was thawed at room temperature. To reduce evaporation and start the assay as rapidly as possible, 200 μL water was added to the perimeter wells to clear-bottomed 96-well plates (Greiner Bio One Cat. No. 655090, Monroe, NC, USA) and the plates were warmed in a 37 °C incubator followed by the addition of 5 μL/well of sample or standard, immediately followed by 70 μL of the assay buffer ± 1 mM DTT (also pre-equilibrated to 37 °C). The assay was initiated by the addition of 75 μL/well of 0.5U 3-PGK enzyme (also pre-equilibrated to 37 °C) as quickly as possible. The plate was transferred to a BioTek Synergy HT plate reader (37 °C) and loss of NADH absorption was measured at 430 nm, at 30 s intervals for 60 min.

### 4.4. Measurement of S-Glutathionylation, Bound G(SH), and Bound G(SS)G to r-GAPDH

IOB-oxidized *r*-GAPDH was prepared exactly as described in Section 4.1. Following the removal of IOB by SCBE, the protein concentrations were determined. Then, the oxidized enzyme was incubated for 15 min with 1:1.2 mol G(SH)/mol *h*-GAPDH subunit followed by incubation with 1 mM G(SH). Both steps were conducted at 37 °C, pH 7. Then, 1 mL of sample was withdrawn from the incubation at time zero and at 15 min intervals for 75 min and was subject to SCBE to remove all non-associated G(SH) from the enzyme. Two aliquots of 0.4 mL sample were added to two YM3 3000 mwco Microcon^®^ centrifugal filters (Millipore Corp, Bedford, MA, USA) followed by 0.1 mL of 0.1 % formic acid/acetonitrile added with rapid pipette mixing to denature the enzyme. After the last time point, the filtration devices were centrifuged at 14,000× *g* at 14 °C for approximately 120 min to for separating the GAPDH subunits (>3000 kDa) in the upper chamber (retentate) from the flow through eluate (lower chamber). The retentates (~15 μL) were reconstituted to 0.5 mL in 0.1% formic acid/acetonitrile. The pairs of eluates’ volumes were measured and pooled. The G(SH) content of S-glutathionylated *r*-GAPDH in the retentate was reduced (G(SH)) and the total bound (G(SS)G + G(SH)) was released following denaturation of the enzyme, and were recovered in the eluates, which were measured using the Promega (Madison, WI, USA) assay kit V6611.

### 4.5. h-GAPDH Active Site Computational Model Methods

The crystal structure of *h*-GAPDH (PDB1u8f) was used as an active site model. The Molecular Operating Environment (MOE) 2020.09 was used to construct, solvate, display, and energetically minimize the model. Merck’s Molecular Force Field (MMFF94s with all MOE parameterization was used with a maximum non-bonded cutoff distance of 12.0Å. The GB/VI generalized solvation model with implicit solvent electrostatics was used [37]. All bound water molecules as determined within the crystal structure were included and immersed in a 6 Å spherical shell of TIP3 waters, and 0.175 M KCl was included for the solvation. The complex was gently relaxed by tethering the backbone, then minimizing to an RMS gradient of 0.05 Å. The addition or modification of bonds within the crystal structure were re-parameterized using the Amber12:EHT force field because of its overall increased accuracy, particularly for small molecules. Non-bonded interaction energies (ΔE) between introduced ligands (G(SH), G(SSH), and C152S-glutathione) and the polypeptide chain were computed in MOE using the Extended Hückel Theory (EHT) hydrogen bond model. The EHT output was sigma (q_σ_) and pi (q_π_) partial charge per atom, along with fractional π bond orders (b_π_). These were used to derive donor (ε_don_) and acceptor (ε_acc_) strengths for interacting atoms and to predict the acidity of the hydrogen bond donors, as well as the basicity of the hydrogen bond acceptors to compute the interaction energy (ΔE). These were combined with scores for the distance and angle between the groups to generate the individual interaction energy ΔE. The sum of all ligand-solvated protein non-bonding interactions yielded an estimate for free energy of the ligand docking process to the protein and served as a useful predictor of ligand binding affinity.

The protein sequence alignment and structure superposition tool in MOE was used to align the native *h*-GAPDH subunit structure with the output from the MDS and energy minimized subunit structures for the C152S-glutathionylated as well as the subunit with G(SS)G docked within the active site. As the aligned proteins had identical primary sequences, the superposition calculations were for a fully populated alignment positions, i.e., alignment positions with no gaps, and the peptide alpha carbon atom was used for the supposition calculations. “The accent secondary structure matches” feature was enabled to increase the weighting of residues with a matching α-helix and β-sheet. The root mean square distance (RMSD) for each alignment column (i.e., residue pair) used during the superposition is represented by a black vertical bar above the pairwise aligned sequences. Closely matching RMSD values are highlighted by a horizontal line cutoff below 2.0 Å, while larger RMSD excursion (5–11 Å) indicates a poor atomic coordinate superposition.

## Figures and Tables

**Table 1:** Total interaction energies (ΔE) between all G(SH), G(SS)G, and NAD^+^ and the local active site polypeptide backbone and side chain atoms within the GAPDH active site region summarized from Supplementary Tables S1 and S2.

Total Ligand Interaction Energies (ΔE kcal/mol)	NAD^+^	G(SH) or G(SS)G
Native GAPDH	−60.5 kcal/mol	-
G(SH) docked in active site with C*_c_*(SH) oxidized to C*_c_*S(OH)	−73.2 kcal/mol	−77.2 kcal/mol
S-glutathionylated C*_c_*(SS)G	−43.0 kcal/mol	−44.3 kcal/mol
G(SH) interactions docked for S_N_2 attack on C*_c_*(SS)G	Not calculated	−55.6 kcal/mol
C*_c_*(SS)G interactions with G(SH) docked for S_N_2 attack by G(SH)	Not calculated	−68.1 kcal/mol
Glutathione disulfide G(SS)G docked in active site	−44.1 kcal/mol	−85.3 kcal/mol

## Data Availability

All data presented in this study are available by written request to P.A.H. (Biochemistry) and M.O.C. Molecular Dynamic Simulations.

## Supplementary Materials

The following supporting information can be downloaded at: https://www.mdpi.com/article/10.3390/ijms24065529/s1.
