# Supplementary material for: Origin of Elevated S-Glutathionylated GAPDH in Chronic Neurodegenerative Diseases
Source: Int J Mol Sci. 2023 Mar 14;24(6):5529. doi: 10.3390/ijms24065529 (PMC10056234; doi:10.3390/ijms24065529)
Supplement: Supplementary file 1 [file ijms-24-05529-s001.zip › ijms-2170727-supplementary.pdf]

Supplementary

Origin of Elevated S-Glutathionylated GAPDH in Chronic Neurodegenerative Diseases

Paul A. Hyslop [1,*], Leonard N. Boggs [1] and Michael O. Chaney [2,†]

[1] Arkley Research Laboratories, Arkley BioTek, LLC, 4444 Decatur Blvd., Indianapolis, IN 46241, USA; lboggs@arkleybiotek.com
[2] Eli Lilly Research Laboratories, Eli Lilly & Co., Lilly Corporate Center, Indianapolis, IN 46285, USA; chaney1643@comcast.net
* Correspondence: physlop@arkleybiotek.com
† Retired.

Keywords: glutathione; oxidative stress; redox signaling; glyceraldehyde−3−phosphate dehydrogenase; hydrogen peroxide; neurodegenerative disease; molecular dynamic simulation

[A] NAD+ Interaction Energies [Native Subunit]				
NAD+	*h*-GAPDH (1U8F)	Type	Bond Length Å	E kcal/mol
O2β 13	Oδ2 ASP 35	H-donor	2.59	-3.4
O2β 17	Oδ1 ASP 35	H-donor	3.16	-0.5
O2β 17	Oδ2 ASP 35	H-donor	2.79	-3.8
C1β 19	Oδ2 ASP 35	H-donor	3.24	-0.6
N6α 27	H_2O	H-donor	3.04	-0.6
N6α 27	H_2O	H-donor	2.92	-2.1
O2δ 52	H_2O	H-donor	2.64	-2
N7N 62	H_2O	H-donor	2.99	-2.6
O1α 2	H_2O	H-acceptor	2.61	-2.8
O1α 2	H_2O	H-acceptor	2.72	-3.2
O1α 2	H_2O	H-acceptor	2.74	-3.2
O2α 3	N ARG 13	H-acceptor	3.51	-1
O2α 3	H_2O	H-acceptor	2.61	-3
O2α 3	H_2O	H-acceptor	2.68	-3.1
O3β 13	H_2O	H-acceptor	2.76	-1.6
O2β 17	H_2O	H-acceptor	2.67	-1.4
N7α 24	H_2O	H-acceptor	2.81	-2.7
N1A 30	H_2O	H-acceptor	2.77	-1.8
O1N 37	H_2O	H-acceptor	2.74	-3.1
O1N 37	H_2O	H-acceptor	2.63	-3
O1N 37	H_2O	H-acceptor	2.85	-2.8
O2N 38	N ILE 14	H-acceptor	2.80	-4.8
O7N 61	N CYS 152	H-acceptor	2.82	-5.3
O7N 61	Sγ CYS 152	H-acceptor	3.05	-0.5
6-ring Cα	ASP 35	π-H	4.90	-0.6
6-ring Cα	ALA 123	π-H	4.75	-1
**Summed Binding Energies (kcal/mol)**				**-60.5**

[B] NAD+ Interaction Energies [GSH →C152(SOH)]				
NAD+	*h*-GAPDH (1U8F)	Type	Bond Length Å	E kcal/mol
O3β 13	Oδ2 ASP 35	H-donor		-1.9
O2β 17	Oδ2 ASP 35	H-donor	2.82	-4.1
O2β 17	Oδ2 ASP 35	H-donor	3.25	-0.5
C1β 19	O THR 99	H-donor	3.28	-0.7
O3D 48	H_2O	H-donor	2.73	-1.9
N7N 62	O ASN 316	H-donor	3.17	-1.9
N7N 62	O GLU 317	H-donor	3.31	-2.7
O1α 2	H_2O	H-acceptor	2.67	-3.1
O1α 2	H_2O	H-acceptor	2.61	-2.7
O1α 2	H_2O	H-acceptor	2.68	-3
O2α 3	H_2O	H-acceptor	2.57	-2.5
O2α 3	H_2O	H-acceptor	2.62	-2.9
O2α 3	H_2O	H-acceptor	2.58	-2.8
O3β 13	O GLY 12	H-acceptor	3.07	-1.3
O3β 13	H_2O	H-acceptor	2.77	-1.5
O2β 17	H_2O	H-acceptor	2.87	-1.2
N7A 24	H_2O	H-acceptor	2.78	-2.8
N1A 30	H_2O	H-acceptor	2.80	-0.9
N3A 33	Cα GLY 10	H-acceptor	3.27	-0.5
O1N 37	N GLY 100	H-acceptor	3.37	-1
O1N 37	Nζ LYS 194	H-acceptor	2.74	-15.6
O1N 37	H_2O	H-acceptor	2.61	-2.5
O2N 38	O THR 182	H-acceptor	2.59	-2.9
O2N 38	H_2O	H-acceptor	3.39	-0.8
O2N 38	H_2O	H-acceptor	2.51	-1.4
O7N 61	N ALA 183	H-acceptor	3.40	-0.8
O7N 61	H_2O	H-acceptor	2.67	-1.8
O1N 37	O LYS 194	Ionic	2.74	-6.5
O2N 38	Nζ LYS 194	Ionic	4.00	-0.5
6-ring	Cα ASP 35	π-H	4.19	-0.5
**Summed Binding Energies (kcal/mol)**				**-73.20**

[C] NAD+ Interaction Energies [C152(SSG)]				
NAD+	*h*-GAPDH (1U8F)	Type	Bond Length Å	E kcal/mol
O3β 13	Oδ2 ASP 35	H-donor	2.61	-3.1
O2β 17	Oδ1 ASP 35	H-donor	2.97	-1.0
O2β 17	Oδ2 ASP 35	H-donor	2.77	-4.0
N6α 27	O ARG 80	H-donor	3.25	-1.4
N6α 27	H_2O	H-donor	3.03	-1.6
O3δ 48	Oγ SER 122	H-donor	2.72	-1.9
C1δ 54	O SER 122	H-donor	3.43	-0.7
C6N 69	O SER 122	H-donor	3.63	-0.7
O1α 2	H_2O	H-acceptor	2.62	-2.8
O2α 3	N ARG 13	H-acceptor	2.86	-5.8
O3β 13	H_2O	H-acceptor	3.00	-1.0
O2β 17	H_2O	H-acceptor	2.73	-1.6
N7α 24	H_2O	H-acceptor	2.77	-2.8
N1α 30	H_2O	H-acceptor	2.89	-1.6
O2N 38	N ILE 14	H-acceptor	2.84	-5.7
O1α 2	H_2O	H-acceptor	2.81	-1.1
O1α 2	H_2O	H-acceptor	2.96	-0.8
O7N 61	Nϵ2 HIS 179	H-acceptor	3.06	-3.3
O7N 61	Nδ2 ASN 316	H-acceptor	2.91	-2.1
**Summed Binding Energies (kcal/mol)**				**-43.0**

[D] NAD+ Interaction Energies [C152SH→ (GSSG)]				
NAD+	*h*-GAPDH (1U8F)	Type	Bond Length Å	E (kcal/mol)
O3β 13	Oδ1 ASP 35	H-donor	2.81	-1.8
O3β 13	Oδ1 ASP 35	H-donor	2.75	-3.6
O2β 17	Oδ1 ASP 35	H-donor	2.73	-2.9
C1β 19	O THR 99	H-donor	3.3	-0.7
N6α 27	O ARG 80	H-donor	2.89	-1.4
O3δ 48	O SER 122	H-donor	2.59	-0.6
C5N 67	Sγ CYS 152	H-donor	3.89	-2
O1α 2	Oγ1 THR 184	H-acceptor	2.72	-3.2
O1α2	H_2O	H-acceptor	2.74	-3.1
O1α 2	H_2O	H-acceptor	3.03	-1.7
O2α 3	N ARG 13	H-acceptor	2.77	-5.2
O2α 3	H_2O	H-acceptor	2.56	-2.3
O5β 4	H_2O	H-acceptor	2.81	-1.4
O3β 13	H_2O	H-acceptor	2.71	-1.5
O2β 17	H_2O	H-acceptor	2.72	-1.5
N7α 24	H_2O	H-acceptor	2.84	-1.8
O3 35	H_2O	H-acceptor	2.84	-1.2
O2N 38	H_2O	H-acceptor	2.61	-3
O3δ 48	Oγ1 THR 103	H-acceptor	3.13	-0.8
O7N 61	N ALA 183	H-acceptor	2.97	-1.6
O7N 61	H_2O	H-acceptor	2.89	-2.1
6-ring	Cα ASP 35	pi-H	4.83	-0.7
**Summed Binding Energies (kcal/mol)**				**-44.1**

Supplementary Table S1. Panel [A]. Interaction energies between NAD^+ and the subunit matrix in the native enzyme. **Panel [B]** Interaction energies between NAD^+ and the subunit matrix after MDS docking of G(SH) within the active site region of the *h*–GAPDH subunit with its catalytic cysteine C152(SH) modified to C152(SOH). **Panel [C]** Interaction energies between NAD^+ and the subunit matrix of the S–glutathionylated subunit. **Panel [D]** Interaction energies between NAD^+ and the subunit matrix after a second molecule of glutathione displaces S–glutathione from the subunit by thiol–disulfide exchange with G(SS)G tightly bound within the active site region.

[A] Top Panel: Interaction Docking of 1st GSH Lower Panel: S-Glutathionylated GAPDH				
Ligand Interaction Energies [C152(SOH) ←G(SH)]				
G(SH)	*h*-GAPDH (1U8F)	Type	Bond (Å)	E kcal/mol
N2 17	Oδ2 ASP 198	H-donor	2.91	-3.7
N3 28	Oδ2 ASP 198	H-donor	3.09	-2.8
N3 28	Oδ1 ASP 198	H-donor	2.89	-5.1
O12 8	Oγ1 THR 153	H-acceptor	2.82	-2.8
O13 8	N GLY 212	H-acceptor	2.85	-5
O31 34	Nζ LYS 194	H-acceptor	2.81	-13.2
O32 34	H_2O	H-acceptor	2.67	-1.7
O32 35	NH1 ARG 197	H-acceptor	2.88	-9.9
O32 35	NH2 ARG 197	H-acceptor	2.75	-9.7
O32 35	H_2O	H-acceptor	2.73	-2.6
O 36	Oγ1 THR 153	H-acceptor	3.06	-0.9
O31 34	Nζ LYS 194	Ionic	2.81	-5.9
O32 35	NH1 ARG 197	Ionic	2.88	-5.4
O32 35	NH2 ARG 197	Ionic	2.75	-6.4
N1 1	5-ring HIS 179	cation-π	3.45	-2.1
**Summed Binding Energies (kcal/mol)**				**-77.2**

Ligand Interaction Energies [C152(SS)G)]				
R(SS)G	*h*-GAPDH (1U8F)	Type	Bond (Å)	E kcal/mol
N1 1	O ALA 213	H-donor	2.81	-2.2
Sγ2 26	Oγ SER 151	H-donor	3.99	-0.6
O12 8	Cα THR 154	H-acceptor	3.37	-1.1
O12 8	N LEU 157	H-acceptor	2.76	-5
O2 22	Oγ1 THR 211	H-acceptor	2.68	-2.4
O31 33	Nϵ ARG 234	H-acceptor	3.00	-2.5
O31 33	NH2 ARG 234	H-acceptor	2.70	-5.2
O31 33	H_2O	H-acceptor	2.73	-3.6
O31 33	H_2O	H-acceptor	2.59	-2.2
O32 34	H_2O	H-acceptor	2.57	-1.1
O32 34	H_2O	H-acceptor	2.57	-1.7
O 35	Cα THR 154	H-acceptor	3.21	-0.6
O 35	Oγ1 THR 154	H-acceptor	2.65	-2.9
Sγ2 26	Oγ SER 151	H-acceptor	3.99	-0.6
Sγ2 26	Oγ SER 151	H-donor	3.99	-0.6
O31 33	Nϵ ARG 234	Ionic	3.00	-4.5
O31 33	NH1 ARG 234	Ionic	2.70	-6.8
O31 34	NH2 ARG 234	Ionic	3.91	-0.7
**Summed Binding Energies (kcal/mol)**				**-44.3**

[B] Top Panel: Docking of 2nd GSH Lower Panel: S-Glutathionylated GAPDH				
Ligand Interaction Energies [C152(SSG) ←G(SH)]				
G(SH)	*h*-GAPDH (1U8F)	Type	Bond (Å)	E kcal/mol
N1 1	Sγ CYS 152	H-donor	3.25	-7.2
N1 1	H_2O	H-donor	2.79	-7.5
N3 27	H_2O	H-donor	3.16	-1.1
O12 8	Oγ SER 151	H-acceptor	2.62	-2.1
O12 8	N G LY 212	H-acceptor	2.71	-6.5
Oϵ1 16	H_2O	H-acceptor	2.72	-2
Sγ2 26	Cβ SER 151	H-acceptor	4.02	-0.5
Sγ2 26	N CYS 152	H-acceptor	3.07	-2.6
O31 33	H_2O	H-acceptor	2.63	-1.5
O31 33	H_2O	H-acceptor	2.59	-3.2
O32 34	Nϵ2 GLN 185	H-acceptor	2.83	-3.5
O32 34	H_2O	H-acceptor	2.64	-2.1
O32 34	H_2O	H-acceptor	2.63	-2.7
O 35	N THR 153	H-acceptor	2.71	-7.4
O 35	Oγ1 THR 153	H-acceptor	2.62	-0.9
O32 34	NH1 ARG 234	Ionic	3.03	-4.3
Cα2 19	5-ring HIS 179	π-H	4.77	-0.5
**Summed Binding Energies (kcal/mol)**				**-55.6**

Ligand Interaction Energies [C152(SSG) ←G(SH)]				
R(SS)G G(SH)	*h*-GAPDH (1U8F)	Type	Bond (Å)	E kcal/mol
N2 17	Oδ2 ASP 198	H-donor	3	-4.8
O12 8	H_2O	H-acceptor	2.57	-1.2
O12 8	H_2O	H-acceptor	2.57	-1.7
O2 22	H_2O	H-acceptor	2.68	-0.8
O2 22	H_2O	H-acceptor	2.71	-2.1
Sγ2 26	H_2O	H-acceptor	2.98	-5.3
O31 33	Nϵ1 TRP 196	H-acceptor	2.88	-3.7
O31 33	NH1 ARG 197	H-acceptor	2.79	-8.9
O31 33	Nζ LYS 215	H-acceptor	2.94	-6.8
O31 33	H_2O	H-acceptor	2.62	-1.9
O32 34	NH2 ARG 194	H-acceptor	2.72	-5.2
O32 34	Nζ LYS 195	H-acceptor	3	-0.5
O32 34	H_2O	H-acceptor	2.68	-3.7
O 35	Cα LYS 197	H-acceptor	3.31	-0.5
O 35	N LEU 197	H-acceptor	2.86	-6.3
O 35	H_2O	H-acceptor	2.57	-2.5
O31 33	NH1 ARG 197	Ionic	2.79	-6.1
O31 33	NH2 ARG 197	Ionic	3.48	-2
O31 33	Nζ LYS 215	Ionic	2.94	-4.9
O32 34	NH1 ARG 197	Ionic	3.34	-2.6
O32 34	NH2 ARG 197	Ionic	2.72	7.9
O32 34	Nζ LYS 215	Ionic	3	-4.5
**Summed Binding Energies (kcal/mol)**				**-68.1**

[C] Interaction Energies of Docked GSSG within active site regionof GAPDH				
Ligand Interaction Energies [C152(SH) → G(SS)G]				
G(SS)G	*h*-GAPDH (1U8F)	Type	Bond (Å)	E kcal/mol
N1 1	Sγ CYS 152	H-donor	2.97	-4.7
N1 1	Oγ1 THR 153	H-donor	3.11	-4.9
N2 17	Oδ1 ASP 198	H-donor	2.96	-4.5
Cβ2 23	Sγ CYS 152	H-donor	3.51	-1.3
N3 27	Oδ1 ASP 198	H-donor	2.74	-6
N3 62	H_2O	H-donor	3.20	-0.6
O12 8	H_2O	H-acceptor	2.60	-1.4
O12 8	H_2O	H-acceptor	2.57	-2.8
O2 22	Oγ1 THR 182	H-acceptor	2.61	-1.4
O32 34	H_2O	H-acceptor	2.64	-1.2
O32 34	H_2O	H-acceptor	2.72	-3.7
O32 34	H_2O	H-acceptor	2.58	-0.5
O32 34	H_2O	H-acceptor	2.66	-3.3
O 35	Oγ SER 151	H-acceptor	2.58	-2.8
O12 43	H_2O	H-acceptor	2.56	-1.4
O12 43	H_2O	H-acceptor	2.55	-2.1
O 51	H_2O	H-acceptor	2.70	-1.3
O2 57	NH1 ARG 197	H-acceptor	3.04	-0.6
O2 57	NH2 ARG 197	H-acceptor	3.04	-1.6
Sγ2 61	N SER 151	H-acceptor	4.23	-1.8
O31 68	Cα SER 125	H-acceptor	3.64	-0.5
O31 68	Nζ LYS 215	H-acceptor	2.81	-2.3
O31 68	H_2O	H-acceptor	2.60	-1.1
O31 68	H_2O	H-acceptor	2.58	-2.6
O32 69	NH1 ARG 197	H-acceptor	2.76	-2.6
O32 69	H_2O	H-acceptor	2.63	-3.1
O 70	N GLY 100	H-acceptor	2.83	-4.1
N1 1	Sγ CYS 152	Ionic	2.97	-4.7
O31 68	NH1 ARG 197	Ionic	3.37	-2.4
O31 68	Nζ LYS 215	Ionic	2.81	-5.8
O32 69	NH1 ARG 197	Ionic	2.76	-6.3
O32 69	NH2 ARG 197	Ionic	3.49	-1.9
**Summed Binding Energies (kcal/mol)**				**-85.3**

Supplementary Table S2. [A]. Top Panel Interaction energy calculations after MDS docking of G(SH) within the active site region of the *h*–GAPDH subunit with its catalytic cysteine C152(SH) modified to C152(SOH). The docked G(SH) ligand aligns poised for nucleophilic attack with its sulfur atom 4.4 Å from the C152(SOH) sulfur atom. **[A]. Bottom Panel** Following formation of the interaction energies between C152S–glutathione moiety and the enzyme weaken. **[B]. Top Panel** Interaction energies of a second molecule of glutathione aligned and docks in an antiparallel aspect to the first C152S–glutathione moiety with its sulfur center within range (4.44 Å) for thiol–disulfide exchange). **[B]. Bottom Panel** the presence of the second docked glutathione molecule weakens the interaction energies between the C152S–glutathione moiety and the enzyme. **[C]** After nucleophilic attack by the docked G(SH) molecule and the C152S–glutathione disulfide, the resulting molecular interactions of the G(SS)G ligand with residues within the active site pocket of the subunit indicate that the G(SS)SG remains tightly bound to the GAPDH inhibiting its dissociation from the enzyme active site. Enhanced residency time of G(SS)G within the active site pocket is likely to be enhanced by the proximity of the sulfur atom of G(SS)G proximal (3.56 Å) to the catalytic sulfur cysteine (see Manuscript Figure 3).

[A] Interaction Energies of docked Cysteine within active site region of GAPDH				
Ligand Interaction Energies [C152(SOH) ←C(SH)]				
C(SH)	*h*-GAPDH (1U8F)	Type	Bond (Å)	E kcal/mol
N1	Sγ CYS 156	H-donor	3.3	-5.9
N1	O SER 292	H-donor	2.78	-6.4
Sγ 10	O HIS 291	H-donor	3.25	-1.4
OXT 13	Oγ1 THR 294	H-acceptor	2.63	-3.4
O 14	Cα TYR 314	H-acceptor	3.36	-0.9
O 14	N ASP 315	H-acceptor	3.03	-3.4
O 14	H_2O	H-acceptor	2.78	-1.2
**Summed Binding Energies (kcal/mol)**				**-22.6**

[B] Interaction Energies of S-cysteinylated GAPDH				
Ligand Interaction Energies [C152(SS)C				
C(SH)	*h*-GAPDH (1U8F)	Type	Bond (Å)	E kcal/mol
N1	O HIS 291	H-donor	2.73	-7
N1	H_2O	H-donor	2.91	-6.7
Sγ 10	Sγ CYS 156	H-donor	4.3	-0.6
OXT 12	N ASP 315	H-acceptor	2.87	-6.0
O 13	N His 291	H-acceptor	3.05	-3.5
**Summed Binding Energies (kcal/mol)**				**-23.8**

[C] Interaction Energies of Docked CSSC within active site region of GAPDH				
Ligand Interaction Energies [C152(SH) → C(SS)C]				
C(SS)C	*h*-GAPDH (1U8F)	Type	Bond (Å)	E kcal/mol
N1	O MET 133	H-donor	2.69	-11.6
N 1	O ASN 155	H-donor	2.75	-7.4
N 1	Oδ1 ASN 155	H-donor	2.76	-8.8
Sγ 10	Sγ CYS 156	H-donor	3.51	-0.8
N 14	SγCYS 156	H-donor	3.3	-1.4
N 14	Oδ2 ASP 315	H-donor	2.71	-18.1
N 14	H_2O	H-donor	2.9	-5.3
Sγ 10	Cα CYS 156	H-acceptor	3.68	-0.6
OXT 12	H_2O	H-acceptor	2.62	-3.4
O 13	NH1 ARG 323	H-acceptor	2.72	-7.1
O 13	H_2O	H-acceptor	2.77	-2.3
O 13	H_2O	H-acceptor	2.62	-2.4
Sγ 23	Cα TYR 320	H-acceptor	3.66	-0.6
OXT 25	N ASP 289	H-acceptor	2.83	-3
OXT 25	Cα GLY 319	H-acceptor	3.39	-0.9
OXT 25	H_2O	H-acceptor	2.56	-2
O 26	N ASP 289	H-acceptor	2.95	-4
O 26	H_2O	H-acceptor	2.64	-3.1
O 13	NH1 ARG 323	Ionic	2.72	-6.7
O 13	NH2 ARG 323	Ionic	3.86	-0.8
N 14	Oδ2 ASP 315	Ionic	2.71	-6.8
**Summed Binding Energies (kcal/mol)**				**-97.1**

Supplementary Table S3. Panel [A]. Interaction energy calculations after MDS docking of cysteine within the active site region of the *h*−GAPDH subunit with its catalytic cysteine C152(SH) modified to C152(SOH). The docked Cysteine ligand aligns poised for nucleophilic attack with its sulfur atom 3.69 Å from the C152(SOH) sulfur atom. **Panel [B].** Interaction energies between S−cysteinylated GAPDH and the subunit polypeptide residues **Panel [C].** After docking a second cysteine molecule within the vicinity of the S−cysteinylated C152 residue, and thiol−disulfide exchange, the resulting strong molecular interactions of the oxidized cystine dipeptide ligand, C(SS)C with residues within the active site pocket subunit indicate that the C(SS)C would remain tightly bound to the GAPDH inhibiting its dissociation from the enzyme active site.

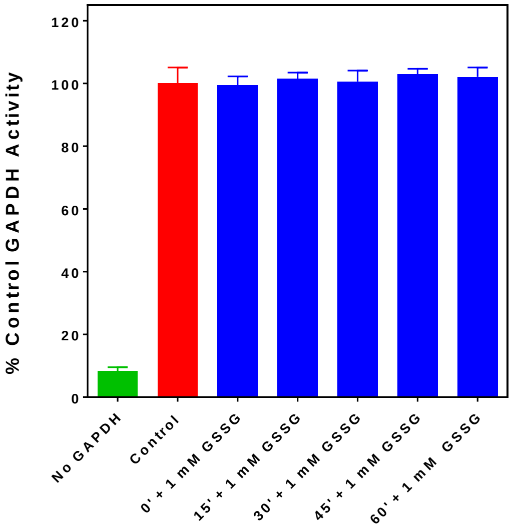

Supplementary Figure S1. Incubation with G(SS)G does not influence GAPDH activity. *r*−GAPDH was incubated with 1 mM G(SS)G for 60 min and then assayed for enzyme activity with 1 mM G(SS)G in the assay buffer (blue bars). The % enzyme activity was not influenced by the presence of GAPDH in the assay buffer compared to activity measured in its absence (red bars).

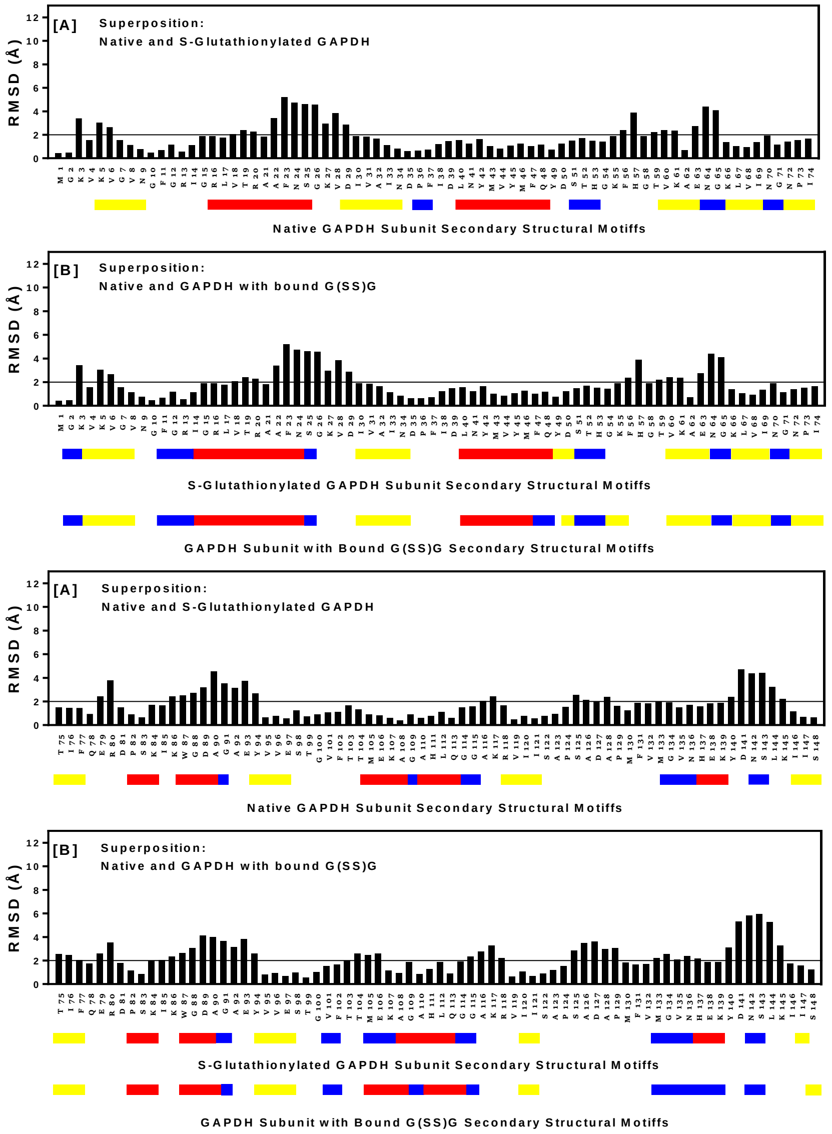

Supplementary Figure S2. *Analysis of residue alignment comparison parameters on the perturbations induced by glutathione binding after MDS* within *the NAD⁺ binding domain (M1-S148) of an h-GAPDH subunit.* The protein sequence alignment and structure superposition tool in MOE is used to align the native *h*-GAPDH subunit structure with the output from MDS, and the energy minimizes subunit structures for C152S-glutathionylated subunit (Panels **[A]**) and the native *h*-GAPDH subunit structure with the output from MDS and the energy minimizes subunit structure for G(SS)G subunit docked within the active site (Panels **[B]**). The root mean square distance (RMSD) for each alignment column (i.e., residue pair) used during the superposition is represented by a black vertical bar above the pairwise aligned sequences. Closely matching RMSD values are highlighted by a horizontal line cutoff below 2.0 Å, while a larger RMSD excursion (5–13 Å) indicates poor atomic coordinate superposition. The linearized secondary structural features for the three structure superpositions are shown below Panels **[A]** (native) and Panels **[B]** (C152S-glutathionylated subunits and subunits with G(SS)G docked within the active site) are color coded as follows: Red α-helix; yellow β-sheet; and blue 2-5 residue turns; while no color is random coil is shown below Panel **[B]** of the RMSD values.

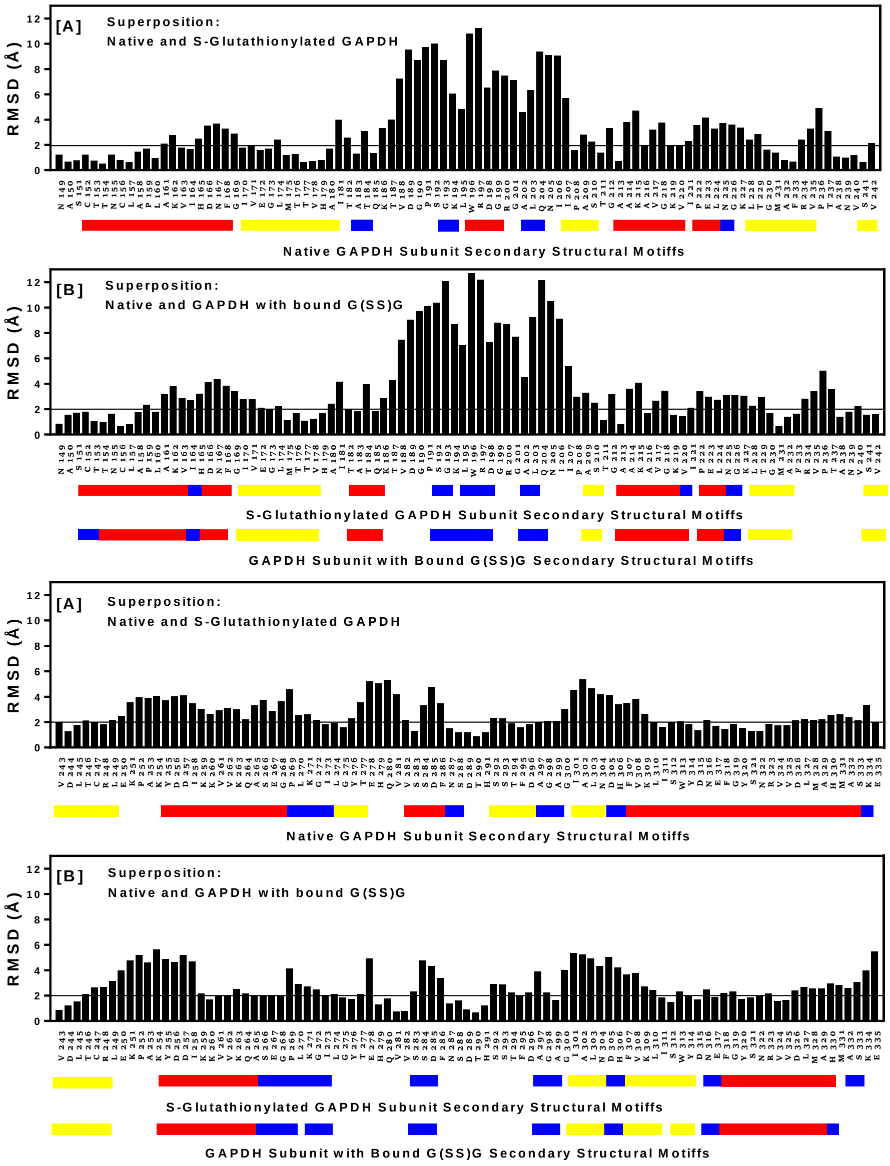

Supplementary Figure S3. *Analysis of residue alignment comparison parameters on the perturbations induced by glutathione binding after MDS* within *the catalytic domain (N149-E335) of an h-GAPDH subunit*. The protein sequence alignment and structure superposition tool in MOE is used to align the native *h*-GAPDH subunit structure with the output from MDS, and the energy minimizes subunit structures for C152S-glutathionylated subunit (Panels **[A]**) and the native *h*-GAPDH subunit structure with the output from MDS and the energy minimizes subunit structure for G(SS)G subunit docked within the active site (Panels **[B]**). The root mean square distance (RMSD) for each alignment column (i.e., residue pair) used during the superposition is represented by a black vertical bar above the pairwise aligned sequences. Closely matching RMSD values are highlighted by a horizontal line cutoff below 2.0 Å, while a larger RMSD excursion (5–13 Å) indicates poor atomic coordinate superposition. The linearized secondary structural features for the three structure superpositions are shown below Panels **[A]** (native enzyme subunits) and Panels **[B]** (C152S-glutathionylated subunits and subunits with G(SS)G docked within the active site) are color coded as follows: Red α-helix; yellow β-sheet; and blue 2-5 residue turns; while no color is random coil is shown below Panel **[B]** of the RMSD values. The region of greatest perturbation and includes all residues in the S-loop region of the subunit (residues 180–203, see text for details).
